# Thermal Oscillation Changes the Liquid-Form Autologous Platelet-Rich Plasma into Paste-Like Form

**DOI:** 10.1155/2022/6496382

**Published:** 2022-05-09

**Authors:** Jean-Lon Chen, Wei-Jen Cheng, Chih-Chi Chen, Shu-Chun Huang, Carl P. C. Chen, Areerat Suputtitada

**Affiliations:** ^1^Department of Physical Medicine & Rehabilitation, Chang Gung Memorial Hospital at Linkou and College of Medicine, Chang Gung University, Guishan District, Taoyuan City, Taiwan; ^2^Department of Rehabilitation Medicine, Faculty of Medicine, Chulalongkorn University, Bangkok, Thailand

## Abstract

**Objectives:**

The texture of the autologous platelet-rich plasma (PRP) that is used in treating degenerative joint diseases such as knee osteoarthritis (OA) is usually in liquid form. However, the turnover rate of protein metabolism in the knee synovial fluid (SF) is less than one hour. This study examined the feasibility of the thermal oscillation technique in converting the liquid-form PRP into an injectable viscous paste-like PRP that may delay the degradation of PRP and continuously release growth factors in the knee joint for a longer period of time.

**Methods:**

This study was conducted in the rehabilitation department of a tertiary hospital. A total of 10 elderly patients with an average age of greater than 65 years and diagnosed with moderate degree of knee OA were recruited. The RegenPRP (RegenLab, Le Mont-sur-Lausanne, Switzerland) test tube chamber was used for PRP generation. A total of 60 milliliters (mL) of blood was drawn from each patient. 10 mL of blood was injected into each PRP test tube chamber. As a result, a total of 6 test tube chambers were obtained and each chamber was centrifuged for 15 minutes. Approximately 5 mL of PRP supernatant (the liquid-form end product) was aspirated and sent for thermal oscillation treatment. Five temperatures were tested: 55, 65, 75, 85, and 95 degrees Celsius. Oscillation was set at 200 revolutions per minute (rpm) for 15 minutes. The enzyme-linked immunosorbent assay (ELISA) was applied in measuring the concentration of platelet-derived growth factor (PDGF) in picogram/milliliter (pg/mL). Repeated measures ANOVA followed by the Bonferroni post hoc test was used to compare the PDGF concentrations between each testing condition.

**Results:**

Under 75 degrees Celsius of heating, the resultant paste-like PRP end product had the highest concentration of PDGF in picograms per milliliter (pg/mL) as compared with other heating conditions (*p* < 0.05). The viscosity of the paste-like PRP was measured to be 70,000 centipoise (cP), which is similar to the viscosity of a toothpaste. The paste-like PRP end product was able to release PDGF continuously for about 14 days, with the highest concentration achieved on the 8^th^ day with an average of 35646 ± 2499 pg/mL. In nonthermally treated liquid-form PRP sample, the highest number of PRP was observed on the 4^th^ day with an average value of 8444 ± 831 pg/mL. Under the heating conditions of 55 and 95 degrees Celsius, the highest concentration of PDGF was observed on the 5^th^ day (13346 ± 764 pg/mL and 3440 ± 303 pg/mL, respectively). Under the heating conditions of 65 and 85 degrees Celsius, the highest concentration of PDGF was observed on the 7^th^ day (15468 ± 744 pg/mL and 20432 ± 1118 pg/mL, respectively).

**Conclusion:**

Through thermal oscillation, liquid-form PRP can be converted to paste-like PRP end product with a viscosity similar to that of a toothpaste. The best heating condition was discovered to be 75 degrees Celsius. The paste-like PRP was able to release PDGF continuously for about 2 weeks, with the highest concentration obtained on the 8^th^ day. The findings in this study suggested that paste-like PRP may be a viable option in treating degenerative knee joint diseases.

## 1. Introduction

Osteoarthritis (OA) is the most common cause of knee pain seen in an orthopedic or rehabilitation outpatient clinic [1, 2]. In knee OA, chondrocytes continue to produce inflammatory mediators that are constantly released into the synovial fluid, resulting in progressive degeneration of the articular cartilage [3, 4]. Autologous platelet-rich plasma (PRP) has been shown to be more effective than other injection options, such as hyaluronic acid (HA), in treating patients with early stages of knee OA [5]. However, the effect of PRP in treating patients with severe stages of knee OA remains controversial [6]. Numerous growth factors are stored in the platelet *α*-granules, such as transforming (TGF-*β*) and platelet-derived growth factors (PDGF). They take active roles in the repair of ligaments, tendons, and articular cartilages [7]. PDGF is a potent growth factor that can be used for cartilage regenerative therapy. It can inhibit the expression of matrix metallopeptidase 9 (MMP-9) and MMP-13 proteins, as well as inhibiting the activation of nuclear factor kappa-light-chain-enhancer of activated B cells (NF-*κ*B) by interleukin-1*β* (IL-1*β*). As a result, apoptosis of chondrocyte cells can be prevented, and the degradation of the cartilage extracellular matrix can be inhibited [8].

The turnover rate of synovial fluid (SF) protein metabolism is about 1 hour. Therefore, most of the SF proteins will probably be metabolized or replaced within a few hours. Particles with higher molecular weights, such as high molecular weight HA, can have a turnover rate greater than 10 hours [9, 10]. When high molecular weight HA is injected into the knee joints, these particles may remain undegraded for a longer period of time. As a result, it is logical to think that when the liquid-form PRP is injected into the knee joint, a certain percentage of growth factors may be degraded or absorbed within a short period of time due to the turnover rate. This may explain why PRP injections are not effective in some knee OA patients.

Studies related to the development of viscous PRP in treating degenerative knee joint disorders are limited [6]. There is one study that mentioned the application of high viscous paste form-like PRP in facial rejuvenation. However, heating PRP to a near-boiling temperature of 95 degrees Celsius may denature the function of PDGF, causing the aggregation of platelets and plasma proteins, yielding a highly viscous platelet-poor plasma (PPP) injectant end product. This PPP can be used as a suitable filler in aesthetic medicine but may not have adequate regenerative capability in treating degenerative disorders [11].

The development of a viscous paste-like PRP injectant may be a feasible option in treating knee OA. It is crucial to establish the right preparatory method such as heating the liquid-form PRP to a temperature of less than boiling point to activate the growth factors without denaturing the platelets. At the same time, simultaneous cell aggregation is also induced, producing the viscous paste-like PRP injectable end product. The resultant product is injectable through 21- or even 23-gauge needle, and after thermal oscillation, the vibrant platelets can release growth factor continuously for a certain period of time. Due to increased viscosity, the paste-like PRP injectant can be contained within the knee joint for a longer period of time and not easily degraded by knee turnover, offering improved soft tissue repair. The hypothesis of this study is that through heating and oscillating the liquid-form PRP, the texture can be changed into paste-like form with increased viscosity. The discovery of a suitable thermal oscillation preparatory method that can increase the viscosity of the liquid-form PRP with the capability of releasing PDGF continuously may be a breakthrough in the treatment of degenerative joint diseases.

## 2. Methods

This study was conducted in the rehabilitation department of a tertiary hospital. A total of 10 elderly patients with an average age of greater than 65 and diagnosed with grade 3 on the Kellgren and Lawrence system (moderate degree of knee OA) for the classification of knee OA were recruited. They were to receive knee intra-articular (IA) PRP injection for the treatment of OA. Blood was drawn from their median cubital veins or cephalic veins for the generation of PRP. Informed consent was obtained from all the participants before participating in this study. This study was approved by the Institutional Reviewer Board (IRB) of Chang Gung Medical Foundation. The IRB number was 202100425A3. All the human research conducted in this study was performed in accordance with the Declaration of Helsinki. Patients taking anticoagulants or with platelet counts less than 150,000 per microliter were excluded from this study. Hematology analysis was done to measure the platelet, neutrophil, lymphocyte, and monocyte counts.

### 2.1. The PRP Testing Conditions

The RegenPRP (RegenLab, Le Mont-sur-Lausanne, Switzerland) test tube chamber for the storage of platelet concentrate was used for PRP generation. Anticoagulant (sodium citrate) is already included inside the chamber. A total of 60 milliliters (mL) of blood was drawn from each patient. 10 mL of blood was injected into each PRP test tube chamber. As a result, a total of 6 test tube chambers were obtained, and each chamber was centrifuged for 15 minutes. The centrifugation speed was set at centrifugal force (RCF) of 1500 × *g* according to the manufacturer's instruction. The RegenPRP test tube chamber yields leukocyte-rich PRP (LR-PRP) with high percentage of mononuclear cell (MNC) recovery [12].

**Figure 1:**
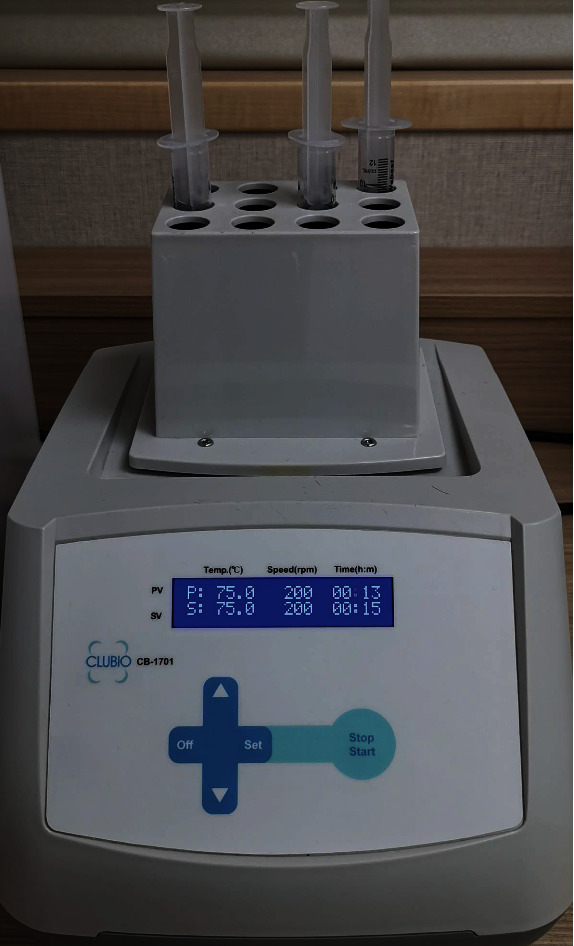
The thermal oscillation technique (machine set at 75 degrees Celsius and under an oscillation speed of 200 revolutions per minute for a total of 15 minutes). Syringe containing the liquid-form PRP was placed into the machine chamber for thermal oscillation. Airtightness is ensured by using the 10 mL syringe.

After centrifugation, the test tube was gently shaken for about 10 times. The RegenPRP test tube chamber has a layer of separator gel that can keep the red blood cell and plasma layers separated. The reason for shaking the test tube was for thorough mixing of the supernatant (the liquid-form end product) as the buffy coat (white blood cells and platelets) may be adhered on top of the gel after centrifugation. Approximately 5 mL of PRP supernatant was aspirated and sent for thermal oscillation treatment. A thermal oscillation machine capable of heating the samples under different temperatures and oscillation speeds (revolutions per minute (rpm)) was used (MEDCLUB SCIENTIFIC Co., Ltd., Taiwan) (Figure 1). The design of this oscillation machine was to ensure that airtightness can be achieved when 10 mL syringe was inserted into the well. The thermal oscillation conditions for the liquid-form PRP samples were as follows:

Condition for chamber 1: not thermally treated—the liquid form PRP end product was sent directly for PDGF concentration measurement.

Condition for chamber 2: thermally treated using 55 degrees Celsius and oscillated under 200 rpm for 15 minutes.

Condition for chamber 3: thermally treated using 65 degrees Celsius and oscillated under 200 rpm for 15 minutes.

Condition for chamber 4: thermally treated using 75 degrees Celsius and oscillated under 200 rpm for 15 minutes.

Condition for chamber 5: thermally treated using 85 degrees Celsius and oscillated under 200 rpm for 15 minutes.

Condition for chamber 6: thermally treated using 95 degrees Celsius and oscillated under 200 rpm for 15 minutes.

After thermal oscillation, daily PDGF concentration (in picogram/milliliter (pg/mL)) was measured for a total of 14 days. During the 14 days of PDGF concentration measurements, the thermal oscillated end product was placed in a beaker with a surrounding temperature of 33 degrees Celsius. This is the average temperature inside the human knee joint [13]. A volume of approximately 250 microliter (*μ*L) of PRP end product was obtained on a daily basis for the measurement of PDGF concentrations. PDGF concentrations were calculated using the appropriate ELISA kits (Cloud-Clone Corporation, USA) in accordance with the manufacturer's instructions [14].

### 2.2. Statistical Analysis

Data analysis was done using the Statistical Program for Social Sciences (SPSS) (SPSS Inc., Chicago, IL) software. The Kolmogorov-Smirnov test was used to determine whether the data obtained in this study was normally distributed or not. Data were expressed as mean and standard deviations (SD). Repeated measures ANOVA followed by the Bonferroni post hoc test was used to observe whether there were significant differences in PDGF concentrations between each sample treatment condition. *p* value of less than 0.05 was considered statistically significant.

## 3. Results

After hematology analysis, the average platelet count was measured to be 202 ± 37 10^9^/L. After centrifugation, platelet count increased by about 1.7 times to 335 ± 61 10^9^/L. After centrifugation, the average neutrophil count decreased by about 68%. The average lymphocyte and monocyte counts increased by about 68% and 50%, respectively (Table 1).

**Figure 2:**
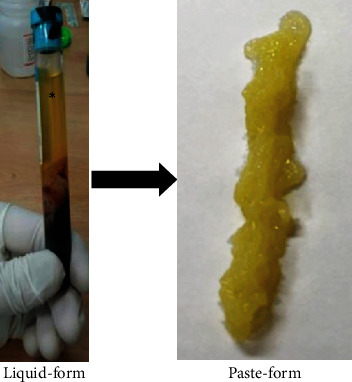
After thermal oscillation using 75 degrees Celsius and under an oscillation speed of 200 revolutions per minute for a total of 15 minutes, the texture of PRP end product changes from liquid form to paste-like form. ∗ indicates the location of the liquid-form PRP in the PRP chamber.

**Figure 3:**
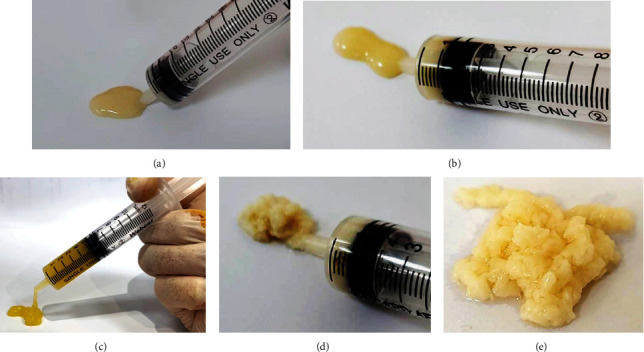
The viscous paste-like PRP end product can be injected through a 21-gauge needle. The appearance and morphology of PRP samples at different temperatures are shown: (a) thermal oscillation using 55 degrees Celsius; (b) thermal oscillation using 65 degrees Celsius; (c) thermal oscillation using 75 degrees Celsius; (d) thermal oscillation using 85 degrees Celsius; (e) thermal oscillation using 95 degrees Celsius.

Result of the Kolmogorov-Smirnov test revealed that the data obtained in the study was normally distributed. When heated to a temperature of 65 degrees Celsius and higher, obvious change in the PRP texture from liquid form into paste-like form would occur (Figure 2). This paste-like PRP end product is injectable using the 23- or 21-gauge needle (Figure 3). At 55 degrees Celsius, the texture of the PRP end product appeared to be semi-liquid.

It was discovered that by heating the liquid-form PRP to a temperature of 75 degrees Celsius under the oscillation speed of 200 rpm, the resultant paste-like PRP end product yielded the highest concentration of PDGF in picograms per milliliter (pg/mL) as compared with other heating conditions (*p* < 0.05, Table 2). The viscosity of the paste-like PRP was measured to be 70,000 centipoise (cP), which is similar to the viscosity of a toothpaste. The paste-like PRP end product was able to release PDGF continuously, and the highest concentration of PDGF was reached on the 8^th^ day for the 75 degrees Celsius thermal oscillation-treated PRP sample with an average of 35646 ± 2499 pg/mL. In the nonthermally treated liquid-form PRP sample, the highest number of PRP was observed on the 4^th^ day with an average value of 8444 ± 831 pg/mL.

The heating condition of 95 degrees Celsius yielded the least PDGF concentrations from day 1 to day 14 as compared with other conditions. On day 14, the 95 degrees Celsius treatment condition revealed the least concentration of PDGF with a value of 223 ± 26 pg/mL.

**Figure 4:**
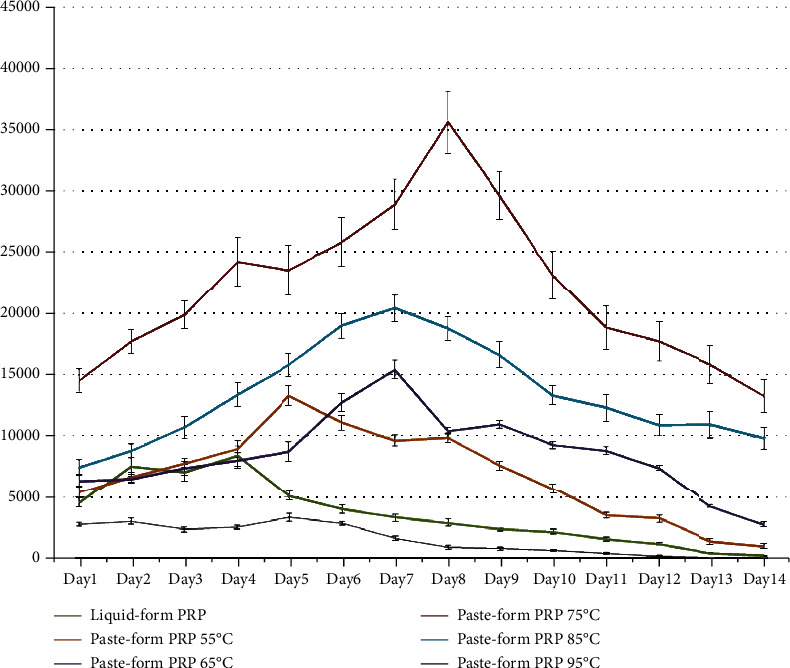
Changes in the PDGF concentrations during the 14-day period after thermal oscillation treatment using different heating temperatures.

Under the PRP treatment conditions of 55 and 95 degrees Celsius, the highest concentration of PDGF was observed on the 5^th^ day. PDGF concentrations were measured to be 13346 ± 764 pg/mL for 55 degrees Celsius and 3440 ± 303 pg/mL for 95 degrees Celsius. For the heating conditions of 65 and 85 degrees Celsius, the highest concentration of PDGF was observed on the 7^th^ day. PDGF concentrations were measured to be 15468 ± 744 pg/mL for 65 degrees Celsius and 20432 ± 1118 pg/mL for 85 degrees Celsius (Figure 4).

## 4. Discussion

This study was aimed at finding the most suitable PRP thermal oscillation treatment condition that can change the texture of PRP from liquid form to paste-like form and at determining which condition can yield the optimal PDGF concentration. The intention of conducting such a study is to generate an injectable highly viscous PRP end product that is not easily degraded by the SF turnover and can release PDGF continuously in the knee joint for a longer period of time. This study has shown that when heating the liquid-form PRP using the temperatures of 65, 75, 85, and 95 degrees Celsius under 200 revolutions per minute (rpm) for 15 minutes, the texture would change from liquid form into paste-like form. The best heating condition was 75 degrees Celsius, which had the highest concentration of PDGF when compared with other temperatures. The highest concentration of PDGF was measured on the 8^th^ day of PRP storage.

In a study by Wen et al., it was discovered that most of the platelet growth factor concentrations increased as each day progresses during the 7-day PRP storage period [15]. In this study, 6 blood samples were used for statistical comparisons. Our study further elaborated on this PRP storage concept by recruiting more blood samples (*N* = 10) and with PDGF concentrations measured up to a period of 14 days. In this study, the LR-PRP with high percentage of mononuclear cell (MNC) recovery was used (RegenPRP test tube chamber) [12]. There are studies suggesting that LR-PRP may fail to alleviate osteoarthritis symptoms due to its inflammatory effects on the synoviocytes [16]. But there are also reports suggesting that the application of LR-PRP can actually benefit the treatment of knee OA [17]. Monocytes and macrophages play a critical role in the development and progression of knee OA. These innate immune cells participate in guiding vascular remodeling and stimulation of local stem cells. Their phenotypes span a continuum of inflammatory (M1) to proregenerative cells (M2). Evidences have shown that subpopulations of monocytes and macrophages stimulate regeneration over inflammatory functions of myeloid cells [17, 18]. In this study, the LR-PRP test tube chambers revealed significant increase in monocyte count after centrifugation, which may have positive implications in the treatment of knee OA.

The autologous plasma injectant with high viscosity is frequently used as suitable fillers in aesthetic medicine [11]. Some studies have tried to combine PRP with hyaluronic acid to increase the viscosity of the injectant [19]. Thermal treatment can increase the viscosity of plasma as the aggregation of platelets and plasma proteins can be promoted. However, high temperatures (ex/exceeding 95 degrees Celsius) can denature the function of PDGF, yielding a platelet-poor plasma (PPP) injectant end product [20].

Perhaps one may question the necessity of developing the thermal oscillation method as PRP may be activated using other strategies such as the addition of calcium chloride [21, 22]. However, some studies have shown that the application of activators such as calcium chloride may not improve the treatment effectiveness of PRP [23]. Therefore, searching for a method that can improve the effectiveness of PRP is needed in treating soft tissue and degenerative disorders. In this study, we have discovered that by heating the liquid-form PRP samples to a temperature above 55 degrees Celsius under the oscillating speed of 200 rpm for 15 minutes, the viscosity of the liquid-form PRP will start to increase. However, at 55 degrees Celsius, the concentration of PDGF is significantly lower as compared with the heating temperatures of 65, 75, and 85 degrees Celsius. When the liquid-form PRP samples were heated to 75 degrees Celsius, obvious paste-like PRP end product can be observed and has a viscosity similar to that of a toothpaste. Under this thermal oscillation condition, it was discovered that the PRP injectant can remain in its viscous paste-like form in an environmental temperature similar to that of the knee joint for approximately 14 days, releasing PDGF continuously during this period of time (Figure 4). Heating must be simultaneously combined with oscillation. Heating without oscillation may result in uneven distribution of the paste-like texture.

Although continuous release of PDGF was also observed in the liquid-form PRP, it is likely to be metabolized in a short period of time as the SF turnover rate in the knee joint is about 1 hour [9]. When the liquid-form PRP was heated up to 95 degrees Celsius, significant decreases in PDGF concentrations were observed in the paste-like PRP end product as compared with other heating temperatures. PDGF was likely to be denatured during the near-boiling point heating process [24]. However, at the heating temperature of 75 degrees Celsius in which the highest PDGF concentration was obtained, it was unlikely that the growth factors were denatured. A study has suggested that heating up to a temperature of 56 degrees Celsius for a period of 1 hour can actually maintain the biological activity of plasma growth factors on ocular surface cells, reducing complement activity and the concentration of immunoglobulin E (IgE) [25].

In our pilot studies, we have injected paste-like PRP injectants (thermally treated using 75 degrees Celsius) into the knee joints of geriatric patients. The Lequesne index was used for the evaluation of treatment effectiveness [26]. Significant improvements in knee pain and function were observed after the injection, lasting up to a period of 6 months. There were no reported adverse effects except possible pain at the injection sites. However, injection-induced pain subsided within 3 days. The shortcoming in this study was that only PDGF was evaluated due to funding limitations. More growth factors will be evaluated (such as vascular endothelial growth factor (VEGF), fibroblast growth factors (FGF), hepatocyte growth factor (HGF), insulin-like growth factor (IGF), epidermal growth factor (EGF), and transforming growth factor (TGF)) in future studies to examine the effect of thermal oscillation on their concentrations. In future studies, the thermal oscillation sample preparation method will also be applied to patients who are taking medications such as steroids and anticoagulants to see if similar increases in growth factor concentrations can be observed as well. Finding a suitable sample preparatory method that can change the texture of liquid-form PRP into a highly viscous paste-like PRP end product that can continuously release PDGF for several days may be a viable option in treating patients with moderate to severe degrees of degenerative knee joint disorders.

## 5. Conclusion

In summary, this study discovered the feasible thermal oscillation sample preparatory method that can change the texture of liquid-form platelet-rich plasma into paste-like form, and continuously release platelet-derived growth factor for a longer period of time. Thermal oscillation under 75 degrees Celsius and 200 revolutions per minute for a total of 15 minutes was shown to be the most suitable preparatory condition as compared with other heating temperatures. Under the laboratory environment that mimics the knee joint temperature, it was shown that the paste-like PRP end product was able to remain undegraded up to a period of about 2 weeks. The results obtained in this study may have significant implications in future PRP-related studies for the treatment of degenerative joint disorders.

## Figures and Tables

**Table 1:** Changes in the percentages of platelets and white blood cells after centrifugation.

Blood cell counts	Status of centrifugation	
	Before centrifugation	After centrifugation
Platelet count (×10^9^/L)	202 ± 37	335 ± 61
Neutrophil count (×10^9^/L)	5.6 ± 1.8	1.8 ± 0.4
Lymphocyte count (×10^9^/L)	3.1 ± 1.1	5.2 ± 1.5
Monocyte count (×10^9^/L)	0.6 ± 0.1	0.9 ± 0.1

Values expressed as mean ± standard deviations (SD). ^∗^*p* < 0.05.

**Table 2:** The changes in the number of platelet-derived growth factors during the 14-day period.

PDGF conc measured each day	Character of PRP					
	Liquid-form PRP (avg from 10 PRP samples)	Paste-like PRP after 55°C thermal oscillation (avg from 10 PRP samples)	Paste-like PRP after 65°C thermal oscillation (avg from 10 PRP samples)	Paste-like PRP after 75°C thermal oscillation (avg from 10 PRP samples)	Paste-like PRP after 85°C thermal oscillation (avg from 10 PRP samples)	Paste-like PRP after 95°C thermal oscillation (avg from 10 PRP samples)
Day 1	4578 ± 358	5468 ± 471	6403 ± 441	14566 ± 944^∗^	7548 ± 564^+^	2885 ± 145
Day 2	7554 ± 744	6692 ± 391	6588 ± 364	17744 ± 991^∗^	8873 ± 589^+^	3112 ± 220
Day 3	7078 ± 678	7856 ± 338	7464 ± 564	19922 ± 1098^∗^	10764 ± 915^+^	2465 ± 201
Day 4	8444 ± 831	8994 ± 663	8065 ± 674	24311 ± 1977^∗^	13444 ± 977^+^	2611 ± 138
Day 5	5147 ± 368	13346 ± 764	8815 ± 787	23566 ± 2087^∗^	15836 ± 989^+^	3440 ± 303
Day 6	4088 ± 309	11155 ± 588	12777 ± 718	25888 ± 1991^∗^	18999 ± 1008^+^	2938 ± 137
Day 7	3399 ± 287	9702 ± 452	15468 ± 744	28974 ± 2041^∗^	20432 ± 1118^+^	1774 ± 155
Day 8	2987 ± 255	9917 ± 408	10506 ± 279	35646 ± 2499^∗^	18774 ± 944^+^	996 ± 166
Day 9	2429 ± 158	7650 ± 346	11057 ± 311	29667 ± 1909^∗^	16646 ± 1066^+^	914 ± 133
Day 10	2266 ± 199	5759 ± 390	9319 ± 277	23168 ± 2009^∗^	13378 ± 778^+^	766 ± 88
Day 11	1677 ± 147	3564 ± 244	8878 ± 315	18860 ± 1742^∗^	12364 ± 1081^+^	534 ± 67
Day 12	1301 ± 108	3311 ± 308	7463 ± 233	17737 ± 1591^∗^	10940 ± 899^+^	331 ± 47
Day 13	583 ± 29	1478 ± 234	4414 ± 144	15912 ± 1549^∗^	11003 ± 1078^+^	228 ± 29
Day 14	361 ± 28	1123 ± 205	2887 ± 189	13337 ± 1367^∗^	9916 ± 865^+^	223 ± 26

Values expressed as mean ± standard deviations (SD). PRP: platelet-rich plasma; conc: concentration; pg/mL: picograms per milliliter (number of PDGF in pg/mL). ^∗^Significantly increased as compared with all other sample treatment conditions (*p* < 0.05). ^+^Significantly increased as compared with all other sample treatment conditions (except when compared with 75°C thermal oscillation (*p* < 0.05)).

## Data Availability

Data and results of statistical analyses are presented in Tables 1 and 2. Raw data may be shared to the readers upon request.
